# Studying the Effects and Competitive Mechanisms of YOYO-1 on the Binding Characteristics of DOX and DNA Molecules Based on Surface-Enhanced Raman Spectroscopy and Molecular Docking Techniques

**DOI:** 10.3390/ijms25073804

**Published:** 2024-03-28

**Authors:** Yanjie Li, Zhiwei Li, Penglun Yun, Dan Sun, Yong Niu, Baoli Yao, Kaige Wang

**Affiliations:** 1 Key Laboratory of Photoelectric Technology of Shaanxi Province, National Center for International Research of Photoelectric Technology & Nano-Functional Materials and Application, Institute of Photonics and Photon-Technology, Northwest University, Xi’an 710127, Chinasund@nwu.edu.cn (D.S.);; 2 State Key Laboratory of Transient Optics and Photonics, Xi’an Institute of Optics and Precision Mechanics, Chinese Academy of Sciences, Xi’an 710119, China

**Keywords:** SERS, molecular docking method, drug molecule, binding site, competitive mechanism

## Abstract

Revealing the interaction mechanisms between anticancer drugs and target DNA molecules at the single-molecule level is a hot research topic in the interdisciplinary fields of biophysical chemistry and pharmaceutical engineering. When fluorescence imaging technology is employed to carry out this kind of research, a knotty problem due to fluorescent dye molecules and drug molecules acting on a DNA molecule simultaneously is encountered. In this paper, based on self-made novel solid active substrates NpAA/(ZnO-ZnCl_2_)/AuNPs, we use a surface-enhanced Raman spectroscopy method, inverted fluorescence microscope technology, and a molecular docking method to investigate the action of the fluorescent dye YOYO-1 and the drug DOX on calf thymus DNA (ctDNA) molecules and the influencing effects and competitive relationships of YOYO-1 on the binding properties of the ctDNA-DOX complex. The interaction sites and modes of action between the YOYO-1 and the ctDNA-DOX complex are systematically examined, and the DOX with the ctDNA-YOYO-1 are compared, and the impact of YOYO-1 on the stability of the ctDNA-DOX complex and the competitive mechanism between DOX and YOYO-1 acting with DNA molecules are elucidated. This study has helpful experimental guidance and a theoretical foundation to expound the mechanism of interaction between drugs and biomolecules at the single-molecule level.

## 1. Introduction

Early detection, diagnosis, and treatment of cancer have become significant challenges that people today need to address together [1]. Among these, chemotherapy is still one of the most effective methods of cancer treatment [2]. Among the various targets of chemotherapeutic drugs, deoxyribonucleic acid (DNA) serves as the material basis for storing and expressing genetic information in living organisms and is considered one of the most effective targets [3]. However, such chemotherapy drugs may have toxicity and other side effects on human organs such as the heart [4]. Clarifying the effects and mechanisms of such drugs on DNA interaction at the single-molecule level remains a key issue for the development of drugs with lower/no toxicity.

The drug doxorubicin (DOX) is a broad-spectrum anthracycline drug known for its significant therapeutic effects in various cancers such as breast cancer, ovarian cancer, and multiple myeloma [5,6,7]. Researchers have conducted extensive studies on the interaction between DOX and DNA, leading to a considerable understanding of its mechanism of action: Zhang et al. discovered through fluorescence spectroscopy that DOX interacts with the DNA bases cytosine and guanine [8]; Wang et al. found through infrared spectroscopy that DOX interacts with DNA phosphate groups through electrostatic interactions [9]; Pérez-Arnaiz et al. found through comparison of equilibrium constants that DOX binds to both A-T and G-C regions [10]; Pei et al. confirmed that DOX binds more readily to DNA at sites such as 5′-GC and 5′-GG [11]; and Zhang et al. demonstrated that DOX not only reacts with the A, T, C, and G bases and phosphate groups on DNA but also affects the spatial configuration of DNA molecules [12]. However, due to its side effects such as cardiotoxicity, gastrointestinal toxicity, and skin toxicity, DOX still faces certain limitations in clinical promotion and application [13].

Generally, methods for studying the interaction between drug molecules and DNA include the following [14,15,16]: atomic force microscopy (AFM), ultraviolet–visible spectroscopy (UV–vis), fluorescence spectroscopy, Fourier transform infrared spectroscopy (FT-IR), and Raman spectroscopy. Among them, Raman spectroscopy is one of the effective tools for precisely identifying and studying the molecular structure of substances. However, the detection sensitivity of Raman spectroscopy is relatively low, which has greatly restricted and hindered its application. Surface-enhanced Raman spectroscopy (SERS), developed based on the rough surfaces of nanoparticles such as gold and silver, effectively enhances the detection sensitivity of Raman spectroscopy. It enables the effective investigation of interactions between drug molecules and biomacromolecules (such as proteins, amino acids, nucleic acids, and nucleotides) at the single-molecule level [17]. SERS has the advantages of high sensitivity, good selectivity, non-destructiveness, and high spatial resolution. Using SERS technology, Ilkhani et al. found that the aromatic ring portion of DOX molecules binds to DNA molecules through insertion, while the amino sugar portion binds to the grooves of DNA molecules [18]. The Wang research group studied the binding mode between the drug Benda and DNA molecules using SERS technology, elucidating the reasons for inducing cell apoptosis [19]. Additionally, single-molecule fluorescence imaging technology can visualize and rapidly monitor changes in the morphology and conformation of biomolecules before and after drug action, thereby enabling the determination and inference of the interaction characteristics between them [20]. The dye YOYO-1 (Oxazole Yellow Homodimer) is one of the most commonly used fluorescent dyes in fluorescence imaging. It is a dimer of YO-PRO-1 and belongs to the cyanine dimers class of fluorescent dyes [21]. The mode of binding between YOYO-1 and DNA is the intercalation between the aromatic rings at both ends and the base pairs of DNA [22,23,24]. YOYO-1 itself does not fluoresce, but it exhibits intense green fluorescence when embedded in DNA [25].

When the interaction between drugs (e.g., DOX) and biological macromolecules (e.g., DNA) is studied by fluorescence imaging technology, whether it is conventional fluorescence imaging technology, laser scanning confocal fluorescence imaging technology [26], or super-resolution fluorescence imaging technology, such as stimulated emission depletion (STED) nanoscopy [27], it is necessary to label samples with fluorescent dyes (e.g., YOYO-1). During experiments, both fluorescent dyes and drug molecules are all bound to DNA molecules [28,29]. As a result, YOYO-1 will have an unavoidable impact on the interaction between the DOX molecule and with the DNA molecule since there may be a competitive relationship between the YOYO-1 molecule and the DOX molecule; therefore, the sensitivity and accuracy of the results of fluorescence imaging are seriously affected.

Recently, Chanseok Lee et al. found that both YOYO-1 and DOX can elongate DNA molecules, and when intercalated molecules are recovered, the partial recovery rate of DOX is lower than that of YOYO-1 and DNA, indicating different degrees of intimacy in the binding of DOX and YOYO-1 molecules to DNA molecules [30]. Vilhelm Müller et al. used two small molecules, YOYO-1 and netropsin, to bind to DNA simultaneously, and found that netropsin prevents regions rich in A and T bases from binding to YOYO-1, and the competitive binding method only requires the simultaneous addition of YOYO-1 and netropsin to DNA without the need for additional steps to remove excess YOYO-1 [31]. Further research and accurate explanations are still needed to understand the mechanism of YOYO-1’s influence on the interaction between DOX and DNA.

In this study, the SERS method based on a novel NpAA/(ZnO-ZnCl_2_)/AuNPs solid substrate developed by our research group, inverted fluorescence microscope technology, and a molecular docking method are employed to investigate the action of YOYO-1 and DOX on calf thymus DNA (ctDNA) molecules and the influencing effects and competitive relationships of YOYO-1 on the binding properties of the ctDNA-DOX complex.

## 2. Results and Discussion

### 2.1. NpAA/(ZnO-ZnCl_2_)/AuNPs Active SERS Solid Substrate

#### 2.1.1. Structural Characteristics

**Figure 1:**
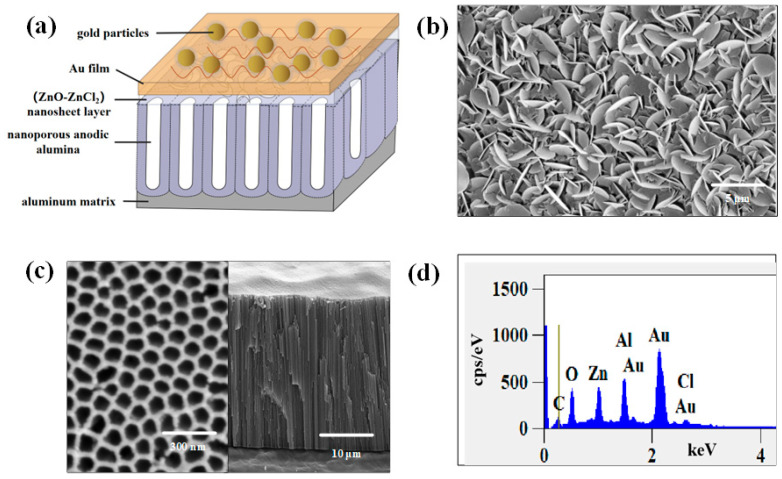
Solid SERS active substrate: (**a**) schematic structure of the NpAA/(ZnO-ZnCl_2_)/AuNPs substrate; (**b**) SEM morphology of the (ZnO-ZnCl_2_) nanosheet layer; (**c**) NpAA template; (**d**) EDS spectrum of the NpAA/(ZnO-ZnCl_2_)/AuNPs substrate.

Figure 1 shows the morphological characterization of the NpAA template and the (ZnO-ZnCl_2_) grown on its surface using scanning electron microscopy (SEM) technology. Figure 1a depicts the schematic structure of the NpAA/(ZnO-ZnCl_2_)/AuNPs substrate, with the aluminum substrate, nanoporous anodic alumina, the (ZnO-ZnCl_2_) nanosheet layer, the gold-coated layer, and gold nanoparticles arranged from bottom to top. Figure 1b,c, respectively, display the SEM morphology of the (ZnO-ZnCl_2_) nanosheet layer and the surface and cross-section of the NpAA template. From Figure 1b, it can be observed that the upper surface of the active solid substrate consists of interconnected and mutually supporting nanosheets, forming an interlaced three-dimensional structure, which increases the number of hotspots. Figure 1c reveals that the overall structure of the NpAA template resembles a honeycomb structure, with the nanopores arranged in an ordered hexagonal array. These vertical channel structures play a significant role in the growth of the (ZnO-ZnCl_2_) nanosheet layer. Figure 1d presents the EDS spectrum of the NpAA/(ZnO-ZnCl_2_)/AuNPs substrate modified with gold nanoparticles, listing the elements in descending order of content, including Au, Al, Zn, Cl, O, and C. The different peaks of the Au element are attributed to the gold film coated on the substrate surface and the modified gold nanoparticles, while the Al element originates from the NpAA template, and the Zn, O, and Cl elements are derived from the (ZnO-ZnCl_2_) nanosheet layer.

#### 2.1.2. SERS Active Performance

**Figure 2:**
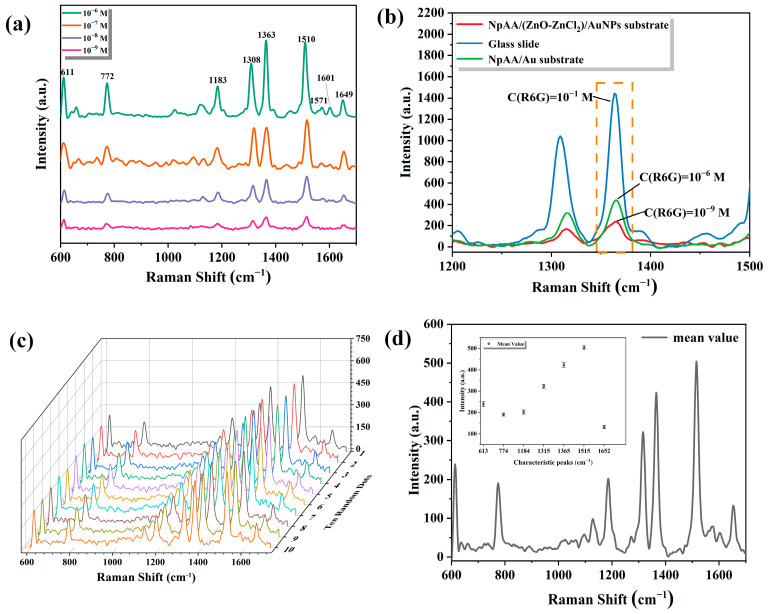
SERS solid substrate NpAA/(ZnO-ZnCl_2_)/AuNPs activity performance: (**a**) sensitivity, C(R6G) = 10^−6^ M~10^−9^ M; (**b**) sensitivity comparison among three substrates (1200~1500 cm^−1^), with the selected peak at 1363 cm^−1^ highlighted in the orange box; (**c**) reproducibility, R6G spectra collected from ten randomly selected spots on the same substrate; (**d**) average spectra of (**c**), inset shows the mean and standard deviation of the intensities of the main peaks, C(R6G) = 10^−8^ M.

Figure 2 shows the results of testing the SERS performance of the NpAA/(ZnO-ZnCl_2_)/AuNPs solid substrate using Rhodamine 6G (R6G) as a probe molecule. The SERS spectra of the R6G solution were used to test the concentration detection limit (sensitivity), enhancement factor, and reproducibility of the active solid substrate. Additionally, the SERS enhancement effect of the NpAA/(ZnO-ZnCl_2_)/AuNPs substrate was compared with that of NpAA/Au and glass slide substrates.

To test the SERS detection limit (sensitivity) of the substrate, the R6G solution was gradually diluted with concentrations of R6G solution ranging from 10^−6^ to 10^−9^ M. The experimental results are shown in Figure 2a. In the figure, the dark green, orange, blue, and purple spectral lines represent the SERS spectra of R6G solutions with concentrations of 10^−6^, 10^−7^, 10^−8^, and 10^−9^ M, respectively, on the solid substrate. From Figure 2a, it can be observed that the characteristic peaks of the R6G solution are mainly concentrated in the range of 500 cm^−1^ to 1700 cm^−1^. Among them, the peak at 611 cm^−1^ is attributed to the bending vibration in the C-C-C ring plane of R6G, the peak at 772 cm^−1^ is attributed to the bending vibration in the C-H plane, the peak at 1183 cm^−1^ is attributed to the stretching vibration of the C-C bond, the peak at 1308 cm^−1^ is attributed to the stretching vibration within the C-N aromatic ring, and the peak at 1363 cm^−1^ is attributed to the stretching vibration of the C-C bond, while 1510 and 1649 cm^−1^ peaks are attributed to the stretching vibration of the aromatic ring’s C-C bonds, and the peak at 1571 cm^−1^ is attributed to the stretching vibration of the benzene ring’s C=C bonds [32,33]. It can be seen that even when the concentration of the R6G solution decreases to 10^−9^ M, the characteristic peak information remains relatively intact.

Figure 2b presents the localized SERS spectra of the R6G solution on the surfaces of three solid substrates. The spectral range depicted is from 1200 cm^−1^ to 1500 cm^−1^. The same volume of R6G solution with concentrations of 10^−1^, 10^−6^, and 10^−9^ M were taken and dropped on slides, NpAA/Au, and NpAA/(ZnO-ZnCl_2_)/AuNPs substrates, respectively, and the three substrates corresponded to the blue, green, and red spectral lines in the graph. The enhancement factor (EF) [34] of the SERS substrates was computed based on the strongest characteristic peak of the R6G solution at 1363 cm^−1^. By rigorously controlling experimental parameters and conditions (such as droplet volume, droplet area, laser excitation area, laser power, exposure times, environmental temperature, etc.), the EF of the substrates was calculated according to Equation (1):

(1) $$EF=\frac{I_{SERS}/C_{SERS}}{I_{Raman}/C_{Raman}}$$

In Equation (1), *I_SERS_* and *I_Raman_* correspond to the peak intensities of the R6G solution at 1363 cm^−1^ on the SERS substrate and the conventional Raman substrate, respectively. *C_SERS_* and *C_Raman_* correspond to the concentrations of the R6G solution in the SERS spectrum and the conventional Raman spectrum, respectively. As observed in Figure 2b, *I_SERS_* is 235 on the NpAA/(ZnO-ZnCl_2_)/AuNPs substrate and 440 on the NpAA/Au substrate, while *I_Raman_* is 1443. Substituting these values into Equation (1), the EF of the NpAA/(ZnO-ZnCl_2_)/AuNPs substrate is approximately 1.63 × 10^7^, and the EF of the NpAA/Au substrate is approximately 3.05 × 10^4^. The NpAA/(ZnO-ZnCl_2_)/AuNPs substrate has a more three-dimensional nanosheet structure and thus has a better enhancement effect.

Figure 2c demonstrates the validation of the repeatability of the SERS signals on the NpAA/(ZnO-ZnCl_2_)/AuNPs substrate by collecting SERS spectral data of the R6G solution (C = 10^−8^ M) from ten random spots on the same solid substrate. From Figure 2c, it can be observed that the differences in the intensities of the characteristic peaks of the R6G solution are minimal, and there is no significant peak shift. The relative standard deviation (RSD) of the intensities of the main characteristic peaks of R6G, describing the repeatability of the NpAA/(ZnO-ZnCl_2_)/AuNPs substrate, is calculated by Equation (2):

(2) $$RSD=\frac{\sqrt{\frac{\sum_{i=1}^{n}{\left(I_{i}-\bar{I}\right)}^{2}}{n-1}}}{\bar{I}}\times 100\%$$

In Equation (2), $I_{i}$ represents the peak intensity of each characteristic peak, n represents the number of randomly collected data, and $\bar{I}$ represents the arithmetic mean intensity of the characteristic peaks. Figure 2d shows the average spectra calculated from Figure 2c, and the inset information is the mean and standard deviation of the peak intensities of the main characteristic peaks of the R6G solution, and the main characteristic peaks obtained from these ten sets of data are within 10%, which indicates that the NpAA/(ZnO-ZnCl_2_)/AuNPs substrate has a better reproducibility.

### 2.2. SERS Spectra

#### 2.2.1. Raman Spectra and SERS Spectra of ctDNA, DOX, and YOYO-1

**Figure 3:**
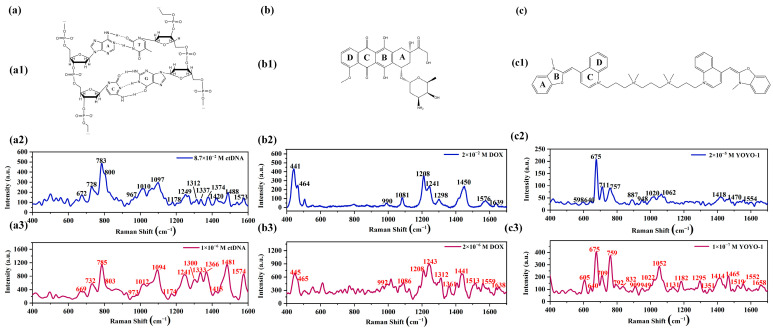
Molecular structures, Raman spectra, and SERS spectra of ctDNA, DOX, and YOYO-1: (**a1**) molecular structure of ctDNA; (**a2**) Raman spectrum of ctDNA solution, 8.7 × 10^−2^ M; (**a3**) SERS spectrum of ctDNA solution, 1 × 10^−6^ M; (**b1**) molecular structure of DOX; (**b2**) Raman spectrum of DOX solution, 2 × 10^−2^ M; (**b3**) SERS spectrum of DOX solution, 2 × 10^−6^ M; (**c1**) molecular structure of YOYO-1; (**c2**) Raman spectrum of YOYO-1 solution, 2 × 10^−5^ M; (**c3**) SERS spectrum of YOYO-1 solution, 1 × 10^−7^ M.

Figure 3 shows the Raman spectra and corresponding SERS spectra of ctDNA, DOX, and YOYO-1 solutions at different concentrations measured using a Raman spectrometer. Molecular structure diagrams of the corresponding samples are provided in the figure to facilitate the understanding of spectral peak positions.

Figure 3(a1) depicts the molecular structure of ctDNA. In Figure 3(a2), the Raman spectrum of the ctDNA solution (8.7 × 10^−2^ M) shows prominent Raman peaks at 672, 728, 783, 800, 967, 1010, 1097, 1178, 1249, 1312, 1337, 1374, 1420, 1488, and 1573 cm^−1^ [12,17,35,36]. The specific peak assignments are as follows: Peaks at 672, 728, 1178, 1249, 1312, 1337, 1374, 1420, 1488, and 1573 cm^−1^ are attributed to vibrations related to the base regions of the ctDNA molecule. Peaks at 783, 800, and 1097 cm^−1^ represent vibrations associated with the phosphate groups. The peak at 1010 cm^−1^ is attributed to the C-O stretching vibration of deoxyribose in double-stranded DNA. Peaks at 672 cm^−1^ and 728 cm^−1^ correspond to the symmetric vibrations of the T and A bases, respectively. The peak at 967 cm^−1^ characterizes deoxyribose in the solution. The peak at 1178 cm^−1^ is attributed to the stretching vibrations of the C-N bond around different bases. The peak at 1249 cm^−1^ is associated with the vibration mode of the C and G bases, while the peak at 1312 cm^−1^ corresponds to the vibration mode of the A and T bases. The peak at 1337 cm^−1^ is attributed to the molecular vibration of the A base, and the peak at 1374 cm^−1^ is associated with the molecular vibration of the A, T, and G bases. Peaks at 1420, 1488, and 1573 cm^−1^ are attributed to the molecular vibrations of the A and G bases. Peaks at 783, 800, and 1097 cm^−1^ correspond to the O-P-O bond in the phosphate group, the main chain, and the O=P=O double bond in PO_2_, respectively.

Figure 3(a3) shows the SERS spectrum of the ctDNA solution (concentration of 10^−6^ M) obtained using NpAA/(ZnO-ZnCl_2_)/AuNPs as the substrate. Compared to the Raman spectrum, the SERS spectrum collected using the active solid substrate not only retains most of the peaks but also eliminates some interference signals caused by non-characteristic peak noise. Compared with conventional Raman spectra, it can be observed that there are slight shifts in multiple peak positions. Among them, the most notable shifts are at 1249 and 1312 cm^−1^, where the characteristic peaks have moved to 1241 and 1300 cm^−1^, respectively. These shifts correspond to the vibrational modes of the bases C and G and A and T, respectively. It is worth noting that the intensities of the characteristic peaks in the range of 1200 cm^−1^ to 1600 cm^−1^ significantly increase in the SERS spectrum. This indicates that certain bases of the ctDNA molecule are closer to the nanosheet layer of the active solid substrate, resulting in better enhancement effects.

Figure 3(b1) depicts the schematic structure of the DOX molecule. Figure 3(b2) shows the Raman spectrum of the DOX solution (2 × 10^−2^ M), with major characteristic peaks at 441, 464, 990, 1081, 1208, 1241, 1576, and 1639 cm^−1^ [37,38]. The specific peak assignments are as follows: the peak at 441 cm^−1^ corresponds to the vibration of the C-C-O bond, the peak at 464 cm^−1^ to the C-O vibration, the peak at 990 cm^−1^ to the C-C vibration in the A ring of the DOX molecule, the peak at 1081 cm^−1^ to the C-O vibration, the peak at 1208 cm^−1^ to the C-O-H vibration, the peak at 1241 cm^−1^ to the vibration of the C-H bond, the peak at 1576 cm^−1^ to the stretching vibration of the C=C bond in the DOX molecule ring, and the peak at 1639 cm^−1^ to the stretching vibration of the C=O bond in the B ring of the DOX molecule. Figure 3(b3) displays the SERS spectrum of the DOX solution (concentration of 2 × 10^−6^ M). A comparison with the Raman spectrum reveals that the SERS spectrum of the DOX solution preserves almost all peak positions and shows more details. Upon comparing the two spectra, it is observed that most peaks exhibit slight shifts.

Figure 3(c1) illustrates the schematic structure of the YOYO-1 molecule. Figure 3(c2) shows the Raman spectrum of the YOYO-1 solution (2 × 10^−5^ M, the solvent is DMSO). The Raman peaks at 640, 675, 711, 948, 1020, and 1418 cm^−1^ are attributed to the DMSO solvent [39,40], where the 675 cm^−1^ peak corresponds to the symmetric stretching of the C-S bond, the 711 cm^−1^ peak to the antisymmetric stretching of the C-S bond, the 948 cm^−1^ peak to CH_3_ rocking, the 1020 cm^−1^ peak to the stretching of the S=O bond, and the 1418 cm^−1^ peak to the deformation of CH_3_. Peaks at 598, 757, 887, 1062, 1470, and 1554 cm^−1^ are attributed to the YOYO-1 molecule [41], where the peak at 598 cm^−1^ corresponds to the twisting of the C ring of the YOYO-1 molecule, the peak at 757 cm^−1^ to the benzimidazole ring, the peak at 887 cm^−1^ to the out-of-plane deformation of the C-H bond, the peak at 1062 cm^−1^ to C-O stretching, the peak at 1470 cm^−1^ to the in-plane deformation of the C-H bond, and the peak at 1554 cm^−1^ to the stretching of the A ring of the YOYO-1 molecule.

Figure 3(c3) presents the SERS spectrum of the YOYO-1 solution (1 × 10^−7^ M, the solvent is DMSO). The peaks attributed to the DMSO solvent in the SERS spectrum are at 640, 675, 709, 949, 1022, and 1414 cm^−1^. Peaks at 605, 759, 792, 832, 909, 1052, 1131, 1182, 1259, and 1552 cm^−1^ are attributed to the YOYO-1 molecule [42,43], where the 605 cm^−1^ peak corresponds to the twisting of the C ring, the 759 cm^−1^ peak to the benzimidazole ring, the 792 cm^−1^ peak to the in-plane deformation of CH_2_, the 832 cm^−1^ peak to C-O stretching, the 909 cm^−1^ peak to the out-of-plane deformation of C-H, the 1052 cm^−1^ peak to C-O stretching, the 1131 cm^−1^ peak to the in-plane deformation of C-H, the 1182 cm^−1^ peak to C-C stretching, the 1259 cm^−1^ peak to the in-plane deformation of CH_2_, and the 1552 cm^−1^ peak to the stretching of the A ring of the YOYO-1 molecule (as shown in Figure 3(c1)). A comparison reveals that the SERS spectrum not only preserves most of the characteristic peaks of the YOYO-1 solution Raman spectrum but also captures some peaks not observed in the Raman spectrum, providing additional detailed information.

#### 2.2.2. SERS Spectrum of YOYO-1 and DOX Mixture

**Figure 4:**
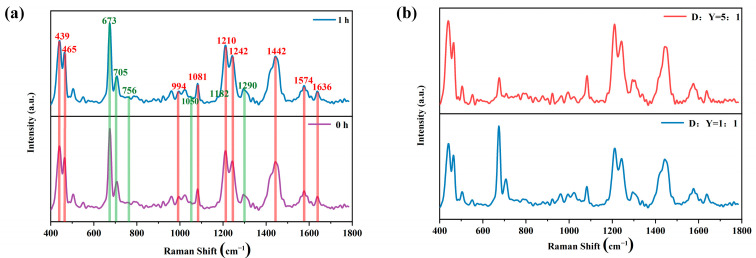
SERS spectra of the YOYO-1 and DOX solution mixture: (**a**) equal molar concentrations with mixing times of 0 h and 1 h; (**b**) mixing time of 1h with molar concentration ratios of 1:1 and 5:1.

Figure 4 shows the SERS spectra of the YOYO-1 and DOX mixture.

To clarify the reaction between DOX molecules and YOYO-1 molecules, the SERS spectra of their mixture were first measured. The purple spectral line in Figure 4a represents the equal volume mixture of DOX solution and YOYO-1 solution at a molar concentration ratio of 1:1, with spectrum information collected after thorough mixing. The blue spectral line represents the spectrum information collected after waiting for one hour in a dark room. In Figure 4a, the main characteristic peaks from the drug molecule DOX and the dye molecule YOYO-1 are labeled and highlighted with red and green numbers and colored bands, respectively. In Figure 4b, the red spectral line represents the spectrum information collected after uniform mixing of DOX solution and YOYO-1 solution at a molar concentration ratio of 5:1, while the blue spectral line represents the spectrum information collected after uniform mixing at a molar concentration ratio of 1:1. From Figure 4a, it can be observed that there is no change in the spectral peaks collected before and after one hour, and the intensities of the characteristic peaks also remain unchanged. In Figure 4b, it can be seen that under consistent experimental conditions except for changing the molar concentration ratio of the two solutions, there is no change in the spectral information, with only slight variations in the intensities of individual characteristic peaks, mainly due to changes in concentration. No new characteristic peaks were generated in either Figure 4a or b. The experimental results fully indicate that there is no reaction between DOX molecules and YOYO-1 molecules.

#### 2.2.3. SERS Spectra of ctDNA-DOX and ctDNA-DOX + YOYO-1

**Figure 5:**
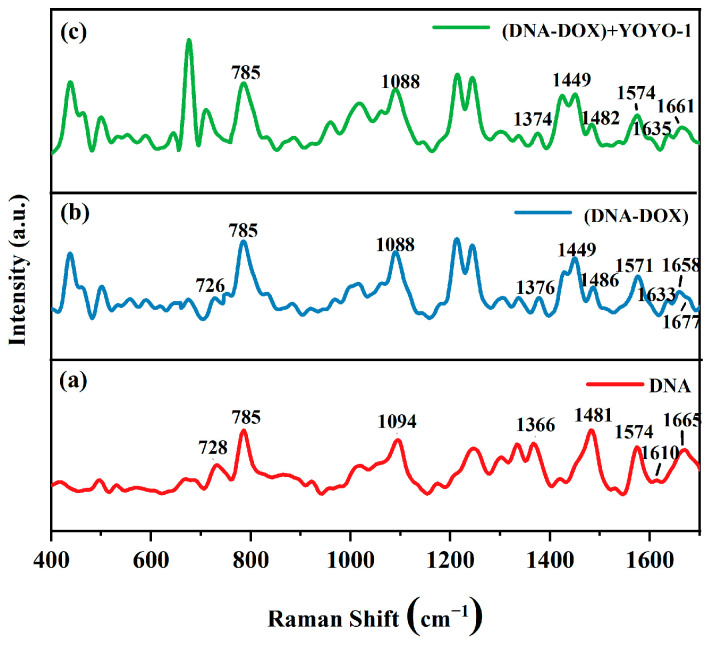
SERS spectra: (**a**) ctDNA solution; (**b**) ctDNA-DOX complex; (**c**) ctDNA-DOX + YOYO-1.

Figure 5 is the control experiment for the SERS spectra illustrating the effect of YOYO-1 on the ctDNA-DOX complex.

The red curve in Figure 5a represents the SERS spectrum of the pure ctDNA solution. The blue curve in Figure 5b represents the SERS spectrum of the ctDNA-DOX complex obtained after thorough mixing of the ctDNA solution with the DOX solution and subsequent incubation for 2 h, with a molar ratio of ctDNA to DOX of 1:2. The green curve in Figure 5c represents the SERS spectrum obtained after adding the YOYO-1 solution to the ctDNA-DOX solution and incubating for 2 h under dark conditions, with a ratio of base pairs of ctDNA to YOYO-1 molecules of 5:1.

The peak assignment and changes in peak positions of various characteristic peaks of the DNA, ctDNA-DOX complexes, and spectra after adding YOYO-1 to ctDNA-DOX complexes are summarized in Table 1.

Comparing the spectra of ctDNA and the ctDNA-DOX complex, changes can be observed in multiple peaks, including those at 728, 1094, 1366, 1481, 1574, 1610, and 1665 cm^−1^. For instance, the peak at 728 cm^−1^ shifted to 726 cm^−1^, corresponding to the symmetric vibration of the A base on ctDNA. The peak at 1094 cm^−1^ shifted to 1088 cm^−1^, attributed to the symmetric vibration of O=P=O in the phosphate group. The peak at 1366 cm^−1^ shifted to 1376 cm^−1^, corresponding to the molecular vibration of base T. The peak at 1481 cm^−1^ shifted to 1486 cm^−1^, attributed to the molecular vibration of base G. The peak at 1574 cm^−1^ shifted to 1571 cm^−1^, corresponding to the molecular vibration of base A. The peak at 1610 cm^−1^ shifted to 1633 cm^−1^, corresponding to the molecular vibration of base A. The peak at 1665 cm^−1^ shifted to 1658 cm^−1^, corresponding to the stretching vibration of C=O in base C. In Figure 5b, there is also an obvious peak change in the range of 1650 to 1685 cm^−1^, and the peak at 1677 cm^−1^ is attributed to hydrogen bonding Watson–Crick complementary pairing, which indicates that the hydrogen bonding between the bases is affected [44]. These shifts indicate interactions between DOX molecules and bases A, T, C, and G, as well as the phosphate group in the DNA chain, revealing the non-specific binding of DOX molecules to ctDNA [12]. Comparing the spectra of ctDNA-DOX complexes and those obtained after adding YOYO-1 solution to the ctDNA-DOX complexes, changes in several peaks, such as those at 1376, 1486, 1571, 1633, and 1658 cm^−1^, can be observed. For example, the peak at 1376 cm^−1^ shifted to 1374 cm^−1^, corresponding to the molecular vibration of base T. The peak at 1486 cm^−1^ shifted to 1482 cm^−1^, attributed to the molecular vibration of base G. The peak at 1571 cm^−1^ shifted to 1574 cm^−1^, corresponding to the molecular vibration of base A. The peak at 1633 cm^−1^ shifted to 1635 cm^−1^, corresponding to the molecular vibration of base A. The peak at 1658 cm^−1^ shifted to 1661 cm^−1^, corresponding to the stretching vibration of C=O in base C. It is evident that only the characteristic peaks of the bases changed, indicating that the YOYO-1 molecules had been inserted between the base pairs of the ctDNA molecule, with few YOYO-1 molecules binding to ctDNA. Additionally, compared to the peaks in the spectra of the ctDNA-DOX complexes, the peaks corresponding to the phosphate group and the DNA chain did not change after the addition of YOYO-1 molecules, indicating that YOYO-1 had not affected the DOX molecules already bound to these sites.

Interaction patterns of some YOYO-1 and DOX molecules with ctDNA molecules are described by the following: When a large amount of DOX molecules is present, they will non-specifically bind to ctDNA molecules, acting on some sites, including A, T, C, and G bases and the phosphate group in the DNA chain. At this point, the addition of YOYO-1 molecules will result in the binding of some YOYO-1 molecules to ctDNA molecules, specifically with YOYO-1 molecules symmetrically inserted between the base pairs of ctDNA molecules. For DOX molecules already bound to phosphate groups and other sites on ctDNA, YOYO-1 will have a minimal effect.

#### 2.2.4. SERS Spectra of ctDNA-YOYO-1 and ctDNA-YOYO-1 + DOX

**Figure 6:**
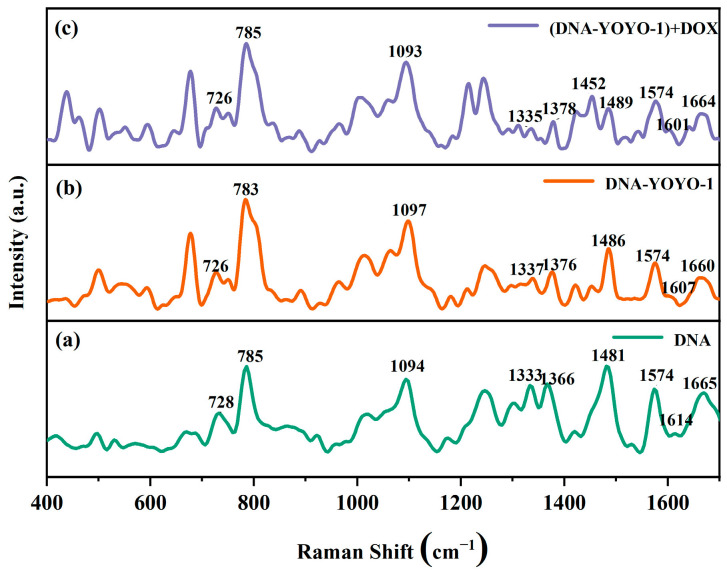
SERS spectra: (**a**) ctDNA solution; (**b**) ctDNA-YOYO-1 complex; (**c**) ctDNA-YOYO-1 + DOX.

Figure 6 shows the control experiment of SERS spectra reflecting the influence of DOX molecules on the ctDNA-YOYO-1 complex.

The dark green curve in Figure 6a represents the SERS spectrum of the ctDNA solution, the orange curve in Figure 6b represents the SERS spectrum of the ctDNA-YOYO-1 complex, and the purple curve in Figure 6c represents the SERS spectrum collected after adding the DOX solution to the ctDNA-YOYO-1 complex. Except for the change in the order of adding the drug and dye, all other experimental conditions remained consistent with the previous ones.

The assignment and changes of characteristic peaks for DNA molecules in the spectra of DNA, the ctDNA-YOYO-1 complex, and mixtures after adding DOX to the ctDNA-YOYO-1 complex are summarized in Table 2.

By comparing the spectra of ctDNA and the ctDNA-YOYO-1 complexes, changes were observed in the characteristic peaks at 728, 785, 1094, 1366, 1481, 1614, and 1665 cm^−1^. Specifically, the peak at 728 cm^−1^ shifted to 726 cm^−1^, attributed to the symmetric vibration of A bases on ctDNA. The peak at 785 cm^−1^ shifted to 783 cm^−1^, associated with the symmetric vibration of the O=P=O in the phosphate group. The peak at 1094 cm^−1^ shifted to 1097 cm^−1^, also related to the symmetric vibration of O=P=O in the phosphate group. The peak at 1366 cm^−1^ shifted to 1376 cm^−1^, attributed to the molecular vibration of T bases. The peak at 1481 cm^−1^ shifted to 1486 cm^−1^, corresponding to the molecular vibration of G bases. The peak at 1614 cm^−1^ shifted to 1607 cm^−1^, associated with the molecular vibration of A bases. The peak at 1665 cm^−1^ shifted to 1660 cm^−1^, attributed to the stretching vibration of C=O in the C bases. These changes indicate that YOYO-1 interacts with A, T, C, and G bases, as well as the phosphate group. Additionally, it interacts with the phosphate group because the process of YOYO-1 molecules inserting between DNA base pairs occurs in two steps [22]: initially, one fluorescent group inserts between the DNA base pairs, creating an intermediate state, while the other fluorescent group at the other end of the YOYO-1 molecule attaches to the phosphate group on the DNA chain. The interaction between YOYO-1 and the phosphate group was not observed when DOX molecules were added first, suggesting that if DOX molecules bind to the DNA chain first, it becomes difficult for YOYO-1 molecules to react with the chain locations on the DNA molecules. Furthermore, comparing the spectra of the ctDNA-YOYO-1 complex and the spectra obtained after adding DOX solution to the ctDNA-YOYO-1 complex revealed significant changes in the characteristic peaks at 783, 1097, 1376, 1486, 1607, and 1660 cm^−1^. Specifically, the peak at 783 cm^−1^ shifted to 785 cm^−1^, attributed to the symmetric vibration of O=P=O in the phosphate group. The peak at 1097 cm^−1^ shifted to 1093 cm^−1^, also related to the symmetric vibration of O=P=O in the phosphate group. The peak at 1376 cm^−1^ shifted to 1378 cm^−1^, attributed to the molecular vibration of T bases. The peak at 1486 cm^−1^ shifted to 1489 cm^−1^, corresponding to the molecular vibration of G bases. The peak at 1607 cm^−1^ shifted to 1601 cm^−1^, associated with the molecular vibration of A bases. The peak at 1660 cm^−1^ shifted to 1664 cm^−1^, attributed to the stretching vibration of C=O in the C bases. These observations indicate strong reactions between DOX molecules and A, T, C, and G bases and the phosphate group. Considering the experimental results shown in Figure 5 and Figure 6, it can be inferred that when the ctDNA-YOYO-1 complex encounters a large amount of DOX molecules, many YOYO-1 molecules are displaced. However, the binding of the ctDNA-DOX complex is more stable; although a small amount of YOYO-1 molecules may be inserted between the DNA base pairs, they cannot displace the DOX molecules bound to the DNA chain.

### 2.3. Fluorescence Experiment

**Figure 7:**
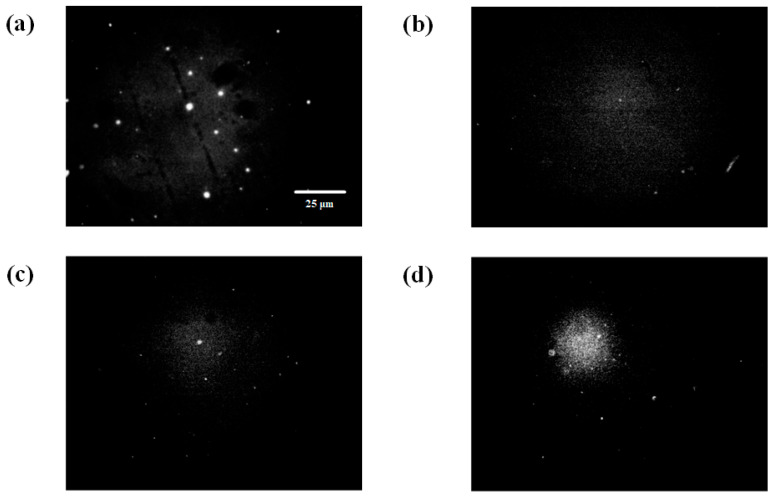
Fluorescence images of the wide field of view for ctDNA-YOYO-1 before and after adding DOX solution: (**a**) fluorescence image of ctDNA-YOYO-1 solution; (**b**) C(DOX) = 1 × 10^−5^ M; (**c**) C(DOX) = 1 × 10^−6^ M; (**d**) C(DOX) = 1 × 10^−7^ M.

Figure 7 illustrates the comparison of the interaction between DOX and the ctDNA-YOYO-1 complex observed using fluorescence microscopy. Figure 7a shows the fluorescence image of the ctDNA-YOYO-1 complex observed by fluorescence microscopy (C(ctDNA) = 1 × 10^−6^ M). Then, a small volume of DOX solution was added to the ctDNA-YOYO-1 complex solution, resulting in final concentrations of DOX in the mixed solution of 1 × 10^−5^ M, 1 × 10^−6^ M, and 1 × 10^−7^ M, respectively. After thoroughly mixing the solution, fluorescence images were captured after waiting for 2 min. Clearly, as the concentration of the DOX solution decreased, as shown in Figure 7b–d, the number of bright spots of ctDNA-YOYO-1 in the fluorescence images increased. Due to the slow decay of the fluorescence intensity of ctDNA-YOYO-1, which does not exceed 10% within 10 min [45], the decrease in the brightness of ctDNA-YOYO-1 and the decrease in the number of spots can be mainly attributed to the detachment of the planar aromatic groups of YOYO-1 molecules from the DNA molecules, thereby no longer emitting light.

This indicates that the more DOX molecules present in the solution, the more YOYO-1 molecules on ctDNA-YOYO-1 will be competitively replaced. This further validates the mechanism of action when both YOYO-1 and DOX molecules act simultaneously on DNA molecules.

### 2.4. Molecular Docking

**Figure 8:**
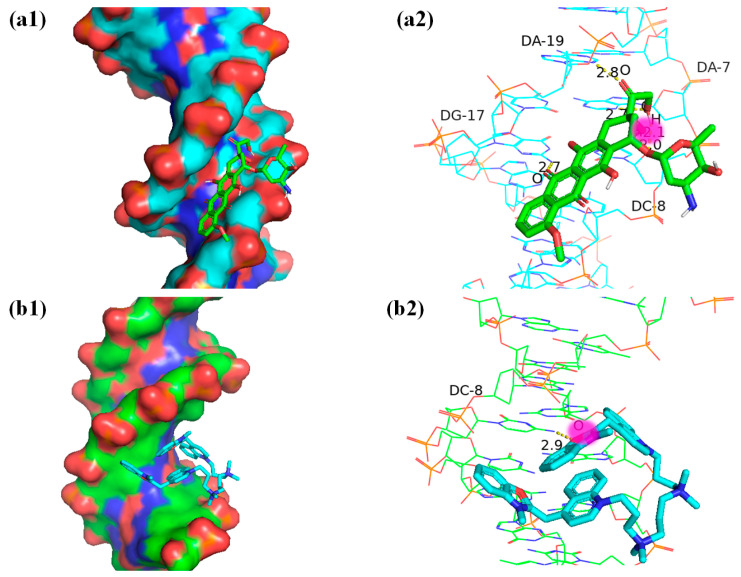
Molecular docking images: (**a1**) DNA-DOX; (**a2**) local details and hydrogen bond length; (**b1**) DNA-YOYO-1; (**b2**) local details and hydrogen bond length.

Figure 8 depicts the docking of DOX, YOYO-1, and DNA molecules.

Using the AutoDock software 4.2.6, 50 random docking simulation experiments were performed with a selected docking box size of 126 × 72 × 72 (x × y × z) and a grid spacing of 0.375 Å. This resulted in 39 clusters of combined conformations for DOX and DNA, with each cluster containing between 1 and 6 different conformations. Figure 8(a1) represents the conformation with the lowest binding energy obtained from the 50 experiments, showing the binding between DOX and DNA. Using the Lamarckian genetic algorithm [46,47], the minimum binding energy between DOX and DNA was calculated as −9.39 kcal/mol, with an average binding energy of −6.91 kcal/mol across the 50 docking experiments. In Figure 8(a2), the local details of the docking site between the DOX molecule and the DNA molecule, as well as the hydrogen bond lengths, are depicted. In this figure, the hydroxyl groups on the right side of ring A (as shown in Figure 3(a2)) are sequentially bound to the A19 and A8 bases, with the oxygen atom between the two hydroxyl groups binding to the C8 base. Additionally, the oxygen atom in ring B is bound to the G17 base. Similarly, molecular docking experiments were performed for YOYO-1 and DNA, also with 50 random trials. The docking box size was 126 × 72 × 72 (x × y × z), and the grid spacing was 0.375 Å. This resulted in a total of 48 clusters of combined conformations for YOYO-1 and DNA, with each cluster containing between 1 and 2 different conformations. Figure 8(b1) illustrates one of the docking results from the 50 trials, showing the insertion of the aromatic rings of YOYO-1 between the base pairs of DNA. Using the Lamarckian genetic algorithm, the minimum binding energy between YOYO-1 and DNA was calculated as −6.92 kcal/mol, with an average binding energy of −3.96 kcal/mol across the 50 docking experiments. In Figure 8(b2), the local details and hydrogen bond lengths of the docking site between the YOYO-1 molecule and the DNA molecule are shown. In the aromatic ring section of YOYO-1, the oxygen atom in the oxazole ring binds to the DNA’s C8 base. Both DOX and YOYO-1 molecules bind to the C8 base, which is one of the competitive binding sites for both molecules.

AutoDock calculates binding energy using computational methods, often based on molecular mechanics force fields and empirical scoring functions. Binding energy is the energy associated with forming a stable complex between molecules, typically a ligand and a receptor. It quantifies the strength of the interaction between the ligand and the receptor, indicating how tightly they bind to each other. A lower binding energy value indicates a stronger and more stable binding between the ligand and the receptor. It can be observed that the binding energy between DOX and DNA is lower than that between YOYO-1 and DNA, indicating a more stable binding between DOX and DNA.

### 2.5. Mechanism of Action

**Figure 9:**
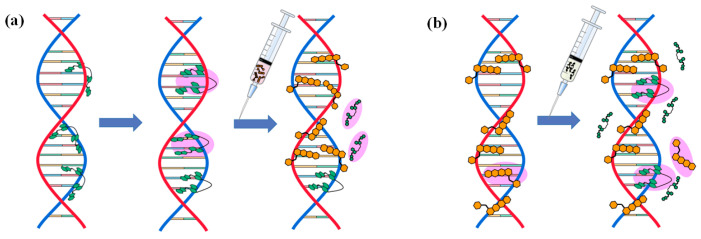
Competitive mechanism model diagram: (**a**) interaction of DOX with ctDNA-YOYO-1 complex; (**b**) interaction of YOYO-1 with ctDNA-DOX complex.

The model of the competitive mechanism when YOYO-1 and DOX molecules act simultaneously on DNA is illustrated in Figure 9. The green molecule represents YOYO-1 and the orange molecule represents DOX. As shown in Figure 9a, when YOYO-1 molecules bind to DNA, it occurs in two steps: first, one end of the aromatic ring inserts between the base pairs, while the other end attaches to the DNA strand, followed by the insertion of the other end between the base pairs on the opposite side, forming a double-insertion layer of YOYO-1 and DNA. Upon the addition of DOX molecules, i.e., the interaction of DOX molecules with ctDNA-YOYO-1 complexes, these DOX molecules replace a large number of YOYO-1 molecules from the DNA, leaving only a small fraction of YOYO-1 molecules on the DNA. As depicted in Figure 9b, when DOX and DNA form the complex ctDNA-DOX, DOX molecules bind non-specifically to DNA, interacting with numerous sites including A, T, C, and G bases, phosphate groups, and the DNA backbone. Upon the addition of YOYO-1 molecules, they insert between the base pairs of DNA. For DOX molecules already bound to sites such as the phosphate group of ctDNA, it is difficult for YOYO-1 to compete with them. When both DOX and YOYO-1 molecules are present simultaneously, DNA molecules tend to preferentially bind with DOX molecules, as the average binding energy between DOX molecules and DNA is significantly lower compared to ctDNA-YOYO-1 molecules. According to the docking results, both DOX and YOYO-1 molecules bind to the C8 base, which serves as a clear competitive binding site.

## 3. Materials and Methods

### 3.1. Materials

The drug DOX used in the experiment was purchased from Sigma-Aldrich (St. Louis, MO, USA). The DOX solution was first configured at a concentration of 2 × 10^−2^ M, and then the DOX solution was diluted to a lower concentration as required for the experiment. The prepared DOX solution was stored in a refrigerator at 4 °C and protected from light. The fluorescent dye YOYO-1 was obtained from Thermo Fisher Scientific (Waltham, MA, USA). The stock solution (concentration of 1 mM) was dissolved in a 0.25% dimethyl sulfoxide (DMSO) solution and stored light-protected at −18 °C in a dry environment. DMSO was purchased from MP Biomedicals, Irvine, CA, USA. The ctDNA used in the experiment was purchased from Sigma-Aldrich. The stock solution of ctDNA (solvent: ultrapure water, concentration: 100 mM) and its diluted concentrations were calculated based on the molar absorption coefficient ε260 = 6600 L/(mol·cm) at a wavelength of 260 nm [48,49]. The drugs Tris HCl (CAS: 1185-53-1) and Bis-Tris (CAS: 6976-37-0) used in the preparation of buffer solutions were purchased from Solarbio (Beijing, China). The buffer solutions were prepared at concentrations of 5 mM, and the solution pH was adjusted to 7.4 and 6.9 using NaOH (5%) and HCl (7%) solutions, respectively. Ultrapure water used in the preparation of solutions was obtained from a Heal Force PTD-20-3 high-purity water instrument. The raw material aluminum foil for the solid substrate was purchased from the Beijing Nonferrous Metal Research Institute (Beijing, China). Anhydrous ethanol, acetone, and oxalic acid reagents were purchased from Xi’an Sanpu Chemical Reagent Co., Ltd. (Xi’an, China), phosphoric acid was obtained from Xiangyang Phosphorus Chemical Co., Ltd. (Xiangyang, China), sulfuric acid was sourced from Shandong Maojun Chemical Co., Ltd. (Zibo, China), and NaOH and ZnCl_2_ were provided by Tianjin Damao Chemical Reagent Factory (Tianjin, China). AuNPs (CAS: 7440-57-5) were purchased from Nanjing XFNANO Materials Tech Co., Ltd. (Nanjing, China), with a diameter of 50 nm and a concentration of 0.05 M, and stored in water. Rhodamine 6G powder was purchased from Sigma-Aldrich. Rhodamine 6G powder was dissolved in ultrapure water to configure a 0.1 M solution of Rhodamine 6G, and then the solution was diluted to the desired concentration.

### 3.2. NpAA/(ZnO-ZnCl_2_)/AuNPs Substrate

The preparation steps of the SERS solid active substrate NpAA/(ZnO-ZnCl_2_)/AuNPs mainly included the following [17,50]: First, the NpAA template substrate was prepared using a standard two-step anodization method. The working conditions for both anodization steps were the same. The first anodization time was 60 min, and the second anodization time was extended to 120 min. Second, the (ZnO-ZnCl_2_) nanosheet layer was formed on the surface of the solid NpAA substrate through self-assembly [51]. A solution of 0.05 M ZnCl_2_ precursor was dripped onto the substrate surface, and then the substrate was sealed and stored at room temperature (25 °C) for one week. Third, a layer of gold film was deposited on the surface of the grown NpAA/(ZnO-ZnCl_2_) using a magnetron sputtering coater, with a thickness of approximately 25nm. If preparing NpAA/Au solid substrate, a gold film was directly deposited on the prepared NpAA surface. Fourth, with the (ZnO-ZnCl_2_) nanosheet layer facing upwards, the entire substrate was immersed in a 5% polyvinylpyrrolidone (PVP) solution in ethanol for 12 h. After rinsing the substrate thoroughly, the surface was modified with 50 nm diameter gold nanoparticles (AuNPs).

### 3.3. Methods

- Surface-enhanced Raman spectrometer

The SERS spectrum acquisition system used self-prepared NpAA/(ZnO-ZnCl_2_)/AuNPs active solid material as the SERS substrate, coupled with the laser confocal Raman system (Alpha 500R, WITec, Ulm, Germany). The system mainly consisted of a laser, single/multi-mode fibers, a spectrograph, a CCD detector, a confocal microscope, and a computer processing module. The solution that was cultivated was placed on the substrate for measurement. A laser with a wavelength of 785 nm was used for the acquisition of solution spectra. A 20× objective lens was used to observe the substrate surface, and a grating of 600 g/mm was used for spectrum acquisition. The exposure time was set to 5 s, and the acquisition was repeated 3 times. The collected raw data not only contained Raman characteristic peaks, but also interference information such as Rayleigh scattering peaks, fluorescence background, cosmic rays, and environmental noise. To reduce the influence of the above factors on the spectral information, we used the WITec Project Four 4.0 software for clipping, cosmic ray removal, baseline correction, and smoothing of the raw data. The final spectral images were plotted using Origin software (Origin 2021). To ensure consistency in experimental conditions, all steps were conducted under constant temperature conditions of 25 °C. The solid substrates for measuring drugs, fluorescent dyes, and DNA molecules were all cut from the same solid base. Each set of spectral data was averaged from 10 valid data points collected at random positions.

- Scanning Electron Microscope (SEM)

The surface morphology of the NpAA template and NpAA/(ZnO-ZnCl_2_) solid substrate was characterized using the Japan Hitachi-su-8010 and CRESTEC-CABL-9000C field emission scanning electron microscopes (Thermo Fisher Scientific, Waltham, MA, USA). Additionally, elemental analysis of the NpAA/(ZnO-ZnCl_2_)/AuNPs substrate after gold nanoparticle modification was performed using an Energy Dispersive X-ray Spectrometer (EDX).

- Inverted Fluorescence Microscope

The experiments were conducted using an inverted fluorescence microscope (IX-70, Olympus, Tokyo, Japan) equipped with an EM-CCD camera (iXon+885, Andor Technology, Belfast, ME, USA). During the experiments, the prepared solutions were placed on coverslips uniformly coated with a layer of poly(methyl methacrylate) (PMMA). A narrow band filter with a center wavelength of 490 nm was used as a filter for the excitation light. A narrow band filter with a center wavelength of 510 nm was used as the filter for the emission light to collect the fluorescence information of YOYO-1. Image processing and analysis of the collected images were performed using ImageJ software (ImageJ2, http://rsbweb.nih.gov/ij/, accessed on 31 December 2023).

- Molecular Docking Software

AutoDock 4.2.6 software (Scripps Research Institute, San Diego, CA, USA) was employed for molecular docking simulations of YOYO-1 and DOX with DNA separately. The software utilizes the Lamarckian genetic algorithm to compute the possible binding conformations and interaction forces. The crystal structure of the DNA receptor (PDB ID: 425D) was obtained from the Protein Data Bank (https://www.rcsb.org/pdb, accessed on 13 September 2023). The three-dimensional structures of the ligands DOX (Compound CID: 31703) and YOYO-1 (Compound CID: 119190) were downloaded from the PubChem database (https://pubchem.ncbi.nlm.nih.gov/, accessed on 14 September 2023). During docking, the receptor macromolecule (i.e., DNA) was kept rigid, and a genetic algorithm was used for random blind docking calculations of the receptor and ligands. The algorithm was run 50 times. Docking results were visualized and analyzed using PyMol 2.5.2 software.

## 4. Conclusions

This paper is based on the surface-enhanced Raman scattering (SERS) spectroscopy technique, utilizing a self-made novel solid active substrate NpAA/(ZnO-ZnCl_2_)/AuNPs in combination with fluorescence imaging technology, molecular docking technology, etc., to thoroughly investigate the competitive mechanism between the fluorescent dye YOYO-1 and the drug DOX when they both interact with DNA molecules. SERS experiments demonstrate that DOX molecules can non-specifically bind to ctDNA molecules, with binding sites including A, T, C, and G bases and phosphate groups. YOYO-1 molecules exhibit dual intercalation with ctDNA molecules; when YOYO-1 molecules interact with the complex ctDNA-DOX, only a small number of YOYO-1 molecules can intercalate between the base pairs of ctDNA molecules without affecting the DOX molecules already bound to the DNA backbone. However, when DOX molecules interact with the complex ctDNA-YOYO-1, a large number of YOYO-1 molecules are replaced by DOX molecules. Molecular docking experiments reveal that DOX and DNA molecules bind to the same bases on DNA, indicating the possibility of competition. Moreover, the average binding energy between DOX and DNA is significantly lower than that between YOYO-1 and DNA. Fluorescence imaging experiments show that when DOX samples are added to the solution of the ctDNA-YOYO-1 complex, the fluorescence brightness of the complex decreases significantly with increasing amounts of DOX, because DOX molecules can replace the YOYO-1 molecules bound to DNA molecules. This study has been helpful in the experimental guidance to expound the mechanism of interaction between drugs and biomolecules at the single-molecule level by combining fluorescence imaging technologies and spectral analysis methods.

## Figures and Tables

**Table 1:** Assignment of characteristic peak positions (600~1700 cm^−1^) for DNA, ctDNA-DOX complexes, and ctDNA-DOX + YOYO-1 mixtures.

ctDNA	ctDNA-DOX	ctDNA-DOX + YOYO-1	Assignment
728	726	/	A
785	785	785	C
1094	1088	1088	O=P=O
1366	1376	1374	T
/	1449	1449	Deoxyribose
1481	1486	1482	G
1574	1571	1574	A
1610	1633	1635	A
1665	1658	1661	C

**Table 2:** Assignment of characteristic peak positions for DNA, the YOYO-1-DNA complex, and the YOYO-1-DNA + DOX mixture (600~1700 cm^−1^).

ctDNA	(ctDNA-YOYO-1)	(ctDNA-YOYO-1) + DOX	Assignment
728	726	726	A
785	783	785	C
1094	1097	1093	O=P=O
1366	1376	1378	T
1481	1486	1489	G
1574	1574	1574	A
1614	1607	1601	A
1665	1660	1664	C

## Data Availability

Data is contained within the article.
